# Biomass-Derived Hard Carbon Anodes for Sodium-Ion Batteries: Recent Advances in Synthesis Strategies

**DOI:** 10.3390/nano15201554

**Published:** 2025-10-12

**Authors:** Narasimharao Kitchamsetti, Kyoung-ho Kim, HyukSu Han, Sungwook Mhin

**Affiliations:** 1 National & Local United Engineering Laboratory for Power Batteries, Faculty of Chemistry, Northeast Normal University, Changchun 130024, China; 2 Department of Semiconductor Equipment Development, Korea Polytechnic University, Anseong-si 17565, Republic of Korea; 3 Department of Energy Science, Sungkyunkwan University, Suwon 16419, Republic of Korea; 4 Department of Energy and Materials Engineering, Dongguk University, Seoul 04620, Republic of Korea

**Keywords:** preparation approaches, biomass, hard carbon, anodes, sodium-ion batteries

## Abstract

Biomass-derived hard carbon (BHC) has attracted considerable attention as a sustainable and cost-effective anode material for sodium-ion batteries (SIBs), owing to its natural abundance, environmental friendliness, and promising electrochemical performance. This review provides a detailed overview of recent progress in the synthesis, structural design, and performance optimization of BHC materials. It encompasses key fabrication routes, such as high-temperature pyrolysis, hydrothermal pretreatment, chemical and physical activation, heteroatom doping, and templating techniques, that have been employed to control pore architecture, defect density, and interlayer spacing. Among these strategies, activation-assisted pyrolysis and heteroatom doping have shown the most significant improvements in sodium (Na) storage capacity and long-term cycling stability. The review further explores the correlations between microstructure and electrochemical behavior, outlines the main challenges limiting large-scale application, and proposes future research directions toward scalable production and integration of BHC anodes in practical SIB systems. Overall, these advancements highlight the strong potential of BHC as a next-generation anode for grid-level and renewable energy storage technologies.

## 1. Introduction

The rapid growth of electric vehicles and portable electronic devices has heightened the demand for energy storage systems with high energy density and economic viability [1]. While lithium-ion batteries (LIBs) remain the dominant technology, their progress is hindered by the scarcity and uneven global distribution of lithium (Li) resources [2]. Furthermore, Li plating on graphite anodes under fast-charging conditions raises safety and durability concerns [3]. These challenges have intensified interest in alternative chemistries for large-scale applications. Sodium-ion batteries (SIBs) are particularly attractive due to the low cost and wide availability of sodium (Na) [4,5]. However, the larger ionic radius of Na^+^ (1.02 Å compared to 0.76 Å for Li^+^) leads to slower diffusion and significant volume fluctuations during cycling, undermining electrode stability and reversibility [6]. Although cathode and electrolyte research has seen substantial progress [7,8], the development of suitable anode materials remains a critical barrier to commercialization.

**Figure 1:**
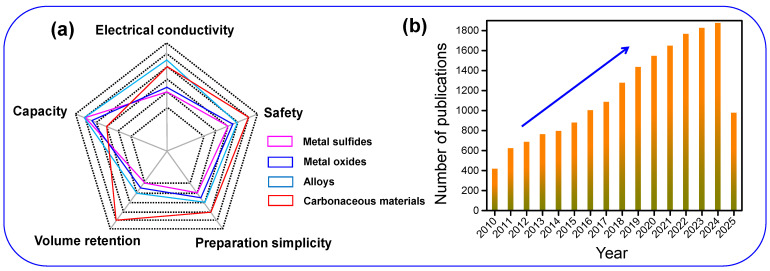
(**a**) Analysis of electrochemical parameters relevant to SIB anode performance. (**b**) Growth in research publications on HC since 2010.

Anode materials are crucial in defining the energy density of SIBs, largely because of their high theoretical capacity. Various candidates have been studied in recent years, including metal oxides [9], sulfides [10], alloys [11], and C-based materials [9], with their comparative performance summarized in Figure 1a. Despite their promising capacities, metal oxides and sulfides generally suffer from poor electrical conductivity, while alloy-based anodes experience severe volume changes during cycling. In contrast, carbonaceous materials demonstrate superior structural integrity and processing advantages. Among them, HC has gained particular attention due to its robust framework, facile synthesis, and desirable electrochemical performance [12]. Its non-graphitized, amorphous structure, comprising randomly oriented graphitic microdomains, enhances Na storage capability and electronic conductivity [13]. Moreover, HC operates at low voltage and offers commercial viability, which is further supported by the increasing volume of related publications (Figure 1b) [14]. Significantly, HC is the first anode material for SIBs to achieve commercial-scale implementation.

A wide range of precursors has been investigated for the preparation of HC. BHCs have gained particular attention owing to their abundance, low cost, and sustainability, making biomass a promising source for HC production [15]. Biomass encompasses diverse renewable organic matter from plants, animals, microorganisms, and their residues, especially agricultural and forestry byproducts [16]. Numerous biomass resources, including sucrose [17], peanut shell [18], rice husk [19], walnut shell [20], pistachio shell [21], apple pomace [22], sorghum stalk [23], pine cone [24], rape seed shuck [25], orange peel [15], banana peel [26], grass [27], wood [28], corncob [29], bamboo [30], loofah [31], coconut shell [32], cucumber stem [33], oak leaf [34], and shaddock peel [35], have been explored as C precursors for HC fabrication. Nevertheless, BHCs often exhibit shortcomings such as low initial coulombic efficiency (ICE) and limited cycling stability [36]. Addressing these challenges requires deeper insights into Na-storage mechanisms and rational engineering of HC microstructures [37,38].

In recent years, significant progress has been reported on various C-based anode materials for SIBs, including heteroatom-doped C [2], porous C derived from agricultural residues [12], and hybrid C composites [30]. While these studies demonstrate notable improvements in electrochemical performance, they often focus on specific synthesis modifications or hybrid approaches. By contrast, comprehensive reviews devoted specifically to BHC synthesis strategies remain scarce. The present review addresses this gap by systematically analyzing recent progress across pyrolysis, hydrothermal, activation, heteroatom doping, and templating approaches, and by outlining the challenges and opportunities for translating BHC anodes into practical SIBs.

**Figure 2:**
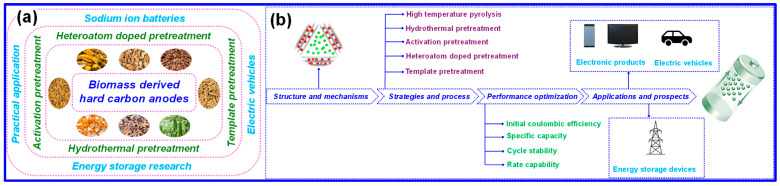
Overview of BHC anodes for SIBs. (**a**) Schematic illustration of structural features and Na^+^ storage mechanisms in BHC. (**b**) Summary of synthesis strategies, performance optimization approaches, and application prospects of BHC anodes in SIBs.

This review begins with an in-depth discussion of the structural characteristics of HC and its Na^+^ storage mechanisms. It then summarizes recent progress in BHC anodes for SIBs, emphasizing synthesis approaches such as high-temperature pyrolysis, hydrothermal treatment, activation, heteroatom incorporation, and template-assisted methods. The final section highlights existing challenges and proposes future research directions for the rational design of next-generation anode materials. Figure 2a illustrates the fundamental structure of HC and the associated Na^+^ storage mechanisms, which serve as the theoretical basis for rational material design. In contrast, Figure 2b summarizes the principal synthesis strategies (hydrothermal, activation, heteroatom doping, and templating), key performance optimization parameters (ICE, cycle stability, specific capacity, rate capability), and practical application prospects in electronic products, electric vehicles, and large-scale energy storage devices.

## 2. Basic Characteristics of HC

This section provides a detailed examination of the structural characteristics of HC and its associated Na storage mechanisms. Such analysis forms the theoretical basis for the rational design and optimization of BHC materials.

### 2.1. Structure of HC

**Figure 3:**
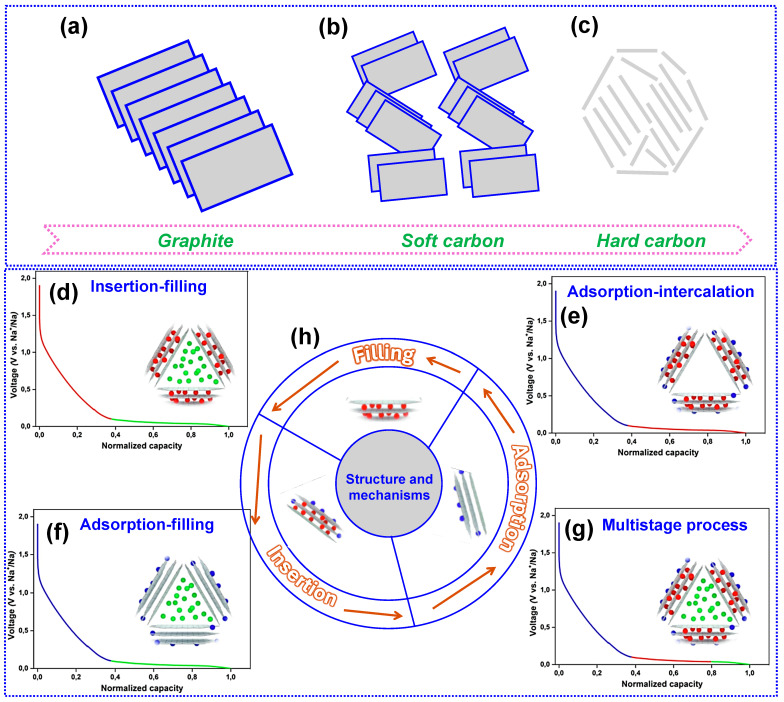
Structures of (**a**) graphite, (**b**) soft C, and (**c**) HC. Four principal models describing the Na storage mechanisms in HC: (**d**) insertion-filling, (**e**) adsorption-insertion, (**f**) adsorption-filling, and (**g**) multistage-process. Adapted from [42]. Copyright 2022, Elsevier B.V. (**h**) Three categories of Na storage in HC.

HC is a non-graphitizable allotrope of C that can endure temperatures up to 3000 °C without undergoing structural transitions [39]. Its unique structural features are highlighted through a comparative analysis with other carbonaceous materials, as depicted in Figure 3a–c. In contrast to the relatively ordered crystalline architectures of graphite and soft C, HC exhibits a highly disordered structure, characterized by increased porosity and a greater density of structural imperfections [40]. The presence of twisted C layers introduces intrinsic voids and enhances interlayer repulsion, thereby expanding the interlayer spacing. The validity of this structural model has been confirmed through multiple experimental approaches. For example, the broad (002) reflection near 22–25° in XRD patterns corresponds to disordered graphitic layers, while Raman D (~1350 cm^−1^) and G (~1580 cm^−1^) bands confirm the presence of structural defects and sp^2^ domains. Scherrer equation-based crystallite size calculations are typically in the range of 1–3 nm, consistent with the nanoscale graphitic domains in BHC. This structural attribute significantly promotes Na-ion storage and diffusion [41]. Consequently, HC demonstrates superior Na storage capacity compared to both graphite and soft C, owing to its porous morphology and enlarged interlayer distances.

### 2.2. Na Storage Mechanism of HC

The mechanisms governing Na storage in HC remain under active investigation, owing to the wide diversity of structural characteristics arising from distinct synthesis methods and precursors. At present, four primary models have been proposed: the “insertion-filling”, the “adsorption-insertion”, the “adsorption-filling”, and the “multistage-process” model [43]. Advancing our understanding of these mechanisms is crucial for the rational design and optimization of HC materials.

#### 2.2.1. Insertion-Filling Mechanism

In 2000, Dahn and colleagues [44] introduced the “insertion-filling” model to explain Na storage in glucose-derived HC. They described HC as a “house of cards” consisting of disordered nanoscale C fragments. In this model, the sloping region of the voltage-capacity profile arises from Na^+^ intercalation between quasi-graphitic layers, while the plateau region reflects Na filling of nanopores (Figure 3d). The model’s applicability to disordered HC was confirmed in 2001 [45]. Further support came from Aniskevich and co-workers [46], who examined commercial HC and observed a two-stage storage process: Na^+^ insertion into graphitic-like domains at higher voltages, indicated by Raman spectral shifts, followed by pore filling at lower voltages, revealed through low-frequency Raman modes associated with Na clustering. These studies collectively validate the “insertion-filling” mechanism.

#### 2.2.2. Adsorption-Insertion Mechanism

Liu’s group [47] in 2012, conducted a pivotal study on polyaniline-based hollow HC nanowires (NWs), observing that Na ion adsorption was predominantly associated with the voltage-sloping region, whereas ion insertion occurred at the voltage plateau, as shown in Figure 3e. This observation formed the basis of the “adsorption-insertion” model. Subsequently, Qiu and collaborators [41] expanded upon this concept, investigating cellulose-derived HC to elucidate the dominant Na storage mechanisms. Their findings demonstrated that a reduction in defect concentration directly impacted the capacity of the sloping region, establishing a linear relationship that strongly supports adsorption as the principal mechanism operative in the voltage-sloping region (C_defect_ + *x* Na^+^ + *x* e^−^ ⇌ Na*_x_*C_defect_).

Furthermore, Cao and co-workers [48] compared HC with graphite and demonstrated that Na storage in HC resembles Li storage in graphite. Their results suggest that the sloping portion of the voltage profile originates from Na^+^ adsorption at defect sites, whereas the low-voltage plateau is associated with Na^+^ insertion and extraction within quasi-graphitic domains. These observations provide strong support for the “adsorption-insertion” model of Na storage in HC.

#### 2.2.3. Adsorption-Filling Mechanism

In 2016, Tarascon and co-workers [49] synthesized carbon nanofibers (CNFs) via electrospinning and systematically modified their surface structural characteristics by carbonizing at various temperatures, thereby demonstrating the validity of the “adsorption-filling” model. Their analysis revealed that Na ions initially adsorb onto defect sites and disordered graphene layers within the voltage-sloping region and subsequently fill the internal pores at the voltage plateau, as depicted in Figure 3f. In a complementary study, Huang’s team [50] fabricated uniform HC microtubes from cotton and examined their structural dynamics during the discharge process. The observation that interlayer spacing remained unchanged indicated a lack of Na ion insertion, thus further corroborating the “adsorption-filling” mechanism.

Additionally, Xu and collaborators [51] applied advanced experimental approaches to probe the relationship between structure and electrochemical behavior in HC. The incorporation of sulfur (S) into the C pore system effectively eliminated the typical low-voltage plateau, providing direct confirmation of a filling-dominated storage mechanism (C_pore_ + *y* Na^+^ + *y* e^−^ ⇌ Na*_y_*C_pore_). Their study further demonstrated that higher carbonization temperatures and fewer structural defects diminished the capacity within the sloping voltage region, consistent with an adsorption-based Na-storage process. Similar Na-ion responses were obtained in both ether- and ester-related electrolytes, corroborating the absence of interlayer insertion. Collectively, these findings strongly validate the proposed “adsorption-filling” mechanism.

#### 2.2.4. Multi-Step Storage Mechanism

Alternative interpretations have suggested that the Na storage mechanism in HC may encompass more than two distinct regions. In 2015, Bommier and collaborators [52] revisited the “house of cards” model and advanced an alternative hypothesis. They posited that Na ions are first inserted into defect sites and subsequently stored through a pore-filling process, thus indicating a multi-step storage pathway.

Building on earlier studies, Alvin and colleagues [53] prepared lignin-derived HC at carbonization temperatures ranging from 1000 to 1500 °C and systematically examined how microstructural characteristics influence Na storage. By conducting detailed analyses of physicochemical properties and Na^+^ ion diffusivity during charge–discharge cycles, they provided an in-depth elucidation of the storage mechanism. The results revealed a multi-stage process beginning with surface adsorption, followed by partial pore filling. At potentials below 0.1 V, Na^+^ insertion into quasi-graphitic domains was observed, while further adsorption in micropores near the cutoff voltage promoted Na clustering. These observations extend beyond the conventional two-region model, as depicted in Figure 3g.

#### 2.2.5. Three Modes of Na^+^ Storage in HC

The operation of SIBs is governed by the reversible shuttling of Na^+^ ions between the cathode and anode, enabling the interconversion of chemical and electrical energy [54,55]. Although the Na storage mechanism in HC is not yet fully resolved, three primary models have been proposed, as illustrated in Figure 3h [56]. These include the following: (1) adsorption of Na^+^ at defect sites on the C surface, (2) intercalation into graphitic interlayers, and (3) confinement within nanopores. Each of these mechanisms is intrinsically influenced by the structural features of HC. Consequently, the rational selection of biomass precursors and the precise control of synthesis parameters are essential for engineering high-performance HC anodes for SIB applications.

## 3. Numerous Preparation Approaches

HC has emerged as a highly promising anode material for SIBs, yet a deeper understanding of its storage mechanisms and targeted improvements remains essential for achieving superior electrochemical performance. As outlined in earlier sections, both its structural features and Na storage characteristics have been extensively studied. Building on this groundwork, the subsequent discussion provides a comprehensive overview of recent developments in BHCs, emphasizing synthesis strategies such as high-temperature pyrolysis, hydrothermal pretreatment, activation, heteroatom incorporation, templating, and other fabrication techniques. Unless otherwise specified, biomass precursors reported in the cited studies were used without further purification, with typical purities exceeding 99% as indicated by the suppliers. Reported synthesis yields for BHC materials generally range between 30 and 45%, depending on the biomass type and processing temperature. Reproducibility was verified across multiple batches in the cited works, and consistent electrochemical performance was observed.

### 3.1. High-Temperature Pyrolysis

High-temperature pyrolysis represents a straightforward thermal decomposition technique wherein biomass precursors are subjected to elevated temperatures under O-free conditions. This process may be further classified into one-step or two-step pyrolysis, contingent upon whether the thermal treatment is conducted in a single continuous stage or in successive phases.

#### 3.1.1. Single-Step High-Temperature Pyrolysis

**Figure 4:**
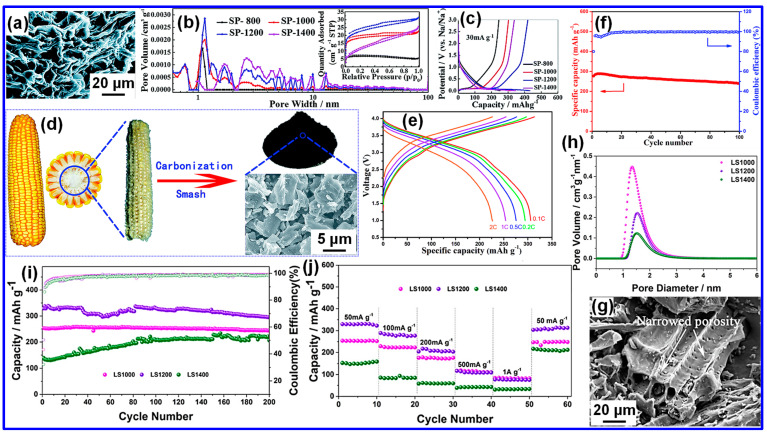
(**a**) SEM micrograph of SP-800. (**b**) Pore size analysis of SP samples calculated via DFT, with the corresponding N_2_ adsorption–desorption behavior displayed in the inset. (**c**) GCD profiles of SP electrodes at 30 mA g^−1^ in Na-ion half-cells. Adapted from [35]. Copyright 2015, Royal Society of Chemistry. (**d**) Illustration of the preparation strategy employed for producing HCC materials. (**e**) GCD curves of a Na_0.9_[Cu_0.22_Fe_0.3_Mn_0.48_]O_2_//HCC1300 full cell at multiple current densities. (**f**) Cycling stability of the full cell at 0.5 C. Adapted from [58]. Copyright 2016, Royal Society of Chemistry. (**g**) SEM micrograph of LS1400. (**h**) Pore size analysis, (**i**) cycling performance at 50 mA g^−1^, and (**j**) rate capability of LS1000, LS1200 and LS1400 samples. Adapted from [59]. Copyright 2019, American Chemical Society.

The one-step high-temperature pyrolysis serves as an important starting point. Xu and colleagues [35] first prepared a series of HC materials from shaddock peel under inert conditions at different pyrolysis temperatures, denoted as SP-T (T representing the temperature). Among these, the SP-800 sample displayed a distinct honeycomb-like structure (Figure 4a), with its pore distribution and N_2_ adsorption/desorption behavior illustrated in Figure 4b. Since no pretreatment was applied, the resulting C exhibited relatively low porosity. Even so, the optimized sample delivered a high reversible capacity of 430 mAh g^−1^ in Na-ion half-cells (Figure 4c), a performance linked to its unique morphology. In a related effort, Chen’s team [57] employed silkworm excrement as the C precursor, producing HC with a comparable honeycomb architecture that achieved 331.7 mAh g^−1^ initially and maintained 258.9 mAh g^−1^ after 100 cycles.

Liu and coworkers [58] investigated corn cobs as a renewable biomass precursor for HC production, with the preparation pathway illustrated in Figure 4d. The optimized sample, HCC1300, was obtained through direct pyrolysis at 1300 °C and demonstrated impressive cycling stability in Na-ion full cells paired with a Na_0.9_[Cu_0.22_Fe_0.3_Mn_0.48_]O_2_ cathode. As illustrated in Figure 4e, the electrode delivered high reversible capacities even under high current conditions. Furthermore, long-term cycling tests (Figure 4f) revealed 92% capacity retention after 100 cycles, underscoring its promise for practical SIB deployment.

These results highlight the potential of corn-cob-derived HC, prepared through one-step pyrolysis, as a practical anode for SIBs. Taken together, the studies reviewed emphasize that a wide variety of biomass feedstocks can be effectively transformed into HC using high-temperature single-step pyrolysis, thereby broadening the range of sustainable anode candidates. Extending this strategy, Wu and colleagues [59] employed lotus seedpods as the C precursor, producing HC samples designated LS1000, LS1200, and LS1400, corresponding to pyrolysis at 1000, 1200, and 1400 °C, respectively. Their analysis revealed a strong dependence of structural characteristics on pyrolysis temperature, with LS1400 exhibiting the tightest interlayer spacing of 3.7 Å, in contrast to 3.91 Å and 3.86 Å for LS1000 and LS1200.

The results clearly demonstrate that increasing the carbonization temperature leads to reduced interlayer spacing and the development of denser pore networks. This trend is confirmed by Figure 4g,h, which display the confined pore structure of LS1400. Additionally, lotus seedpod-derived HC exhibited superior cycling durability (Figure 4i) and impressive rate capability (Figure 4j) in Na-ion half-cells, underscoring its promise as an advanced anode for SIBs. Overall, the study highlights the decisive role of carbonization temperature in governing both the microstructure and electrochemical behavior of HC.

A growing body of research highlights the wide utility of one-step high-temperature treatment for producing HC. For example, Xiao and co-workers [60] prepared HC from pine pollen, which delivered a discharge capacity of 370.1 mAh g^−1^ at 0.1 A g^−1^ in Na-ion half-cells. Similarly, Dong and co-workers [61] synthesized HC from *Ganoderma lucidum* residue, achieving remarkable long-term stability with nearly complete capacity retention after 1500 cycles at 5 A g^−1^. Together, these studies underscore the robustness of single-step pyrolysis as a route for developing high-performance HCs. Through strategic precursor selection and optimization of synthesis parameters, advanced anode materials with strong promise for practical SIBs can be realized.

#### 3.1.2. Two-Step High-Temperature Pyrolysis

Two-step high-temperature pyrolysis improves upon the conventional single-stage process by adding a secondary thermal treatment. The procedure usually begins with low-temperature carbonization and is followed by high-temperature pyrolysis. This staged approach enables better tuning of the pore structure, resulting in enhanced electrochemical behavior and superior performance of HC in energy storage devices.

**Figure 5:**
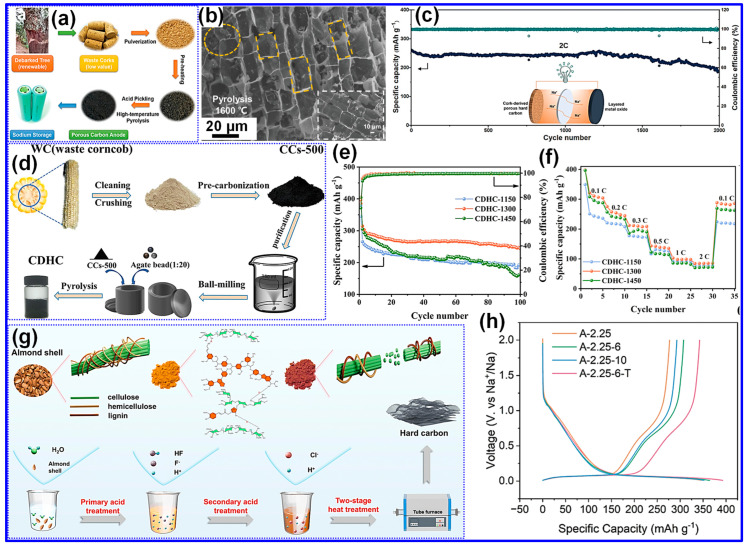
(**a**) Diagram depicting the synthetic pathway of CC materials. (**b**) SEM micrograph of CC-1600. (**c**) Long-term cycling behavior of Na[Cu_1/9_Ni_2/9_Fe_1/3_Mn_1/3_]O_2_ paired with CC-1600 in full-cell configuration under 2C conditions. Adapted from [62]. Copyright 2019, Wiley VCH. (**d**) Diagram depicting the stepwise synthesis procedure of CDHC from corncob biomass. (**e**) Cycle stability at 0.1 C, and (**f**) Rate performance from 0.1 to 2 C of CDHC samples. Adapted from [63]. Copyright 2024, American Chemical Society. (**g**) Illustration outlining the preparation process, and (**h**) Initial discharge/charge profiles at 30 mA g^−1^ of A-2.25, A-2.25-6, A-2.25-10, and A-2.25-6-T samples. Adapted from [64]. Copyright 2024, Royal Society of Chemistry.

In their research, Hu and co-workers [62] employed a two-step high-temperature pyrolysis approach to synthesize HC from waste cork stoppers, as schematically represented in Figure 5a. The initial carbonization was conducted at 800 °C for 2 h, producing the intermediate material CC-Pre-800. This was followed by a second thermal treatment at 1200, 1400, and 1600 °C, respectively, each for 2 h, resulting in samples designated CC-1200, CC-1400, and CC-1600. As illustrated in Figure 5b, carbonization at 1600 °C led to a transformation from the original honeycomb morphology to well-aligned rectangular pore channels with slightly increased diameters, indicating substantial internal structural evolution. The optimal sample demonstrated outstanding cycling stability in Na-ion full cells (Figure 5c), affirming its potential as a high-performance anode for SIBs.

Wang and colleagues [63] reported the synthesis of HC from waste corncobs via a two-step high-temperature pyrolysis strategy (Figure 5d). The procedure involved a primary carbonization at 500 °C for 2 h, followed by ball milling of the intermediate product. A subsequent carbonization stage was then carried out at 1150, 1300, or 1450 °C for 2 h, yielding samples labeled CDHC-1150, CDHC-1300, and CDHC-1450. Among all, CDHC-1300 displayed the most promising electrochemical characteristics, delivering a stable reversible capacity of around 250 mAh g^−1^ after 100 cycles (Figure 5e). It also exhibited strong rate capability (Figure 5f), with an average discharge capacity of 280 mAh g^−1^ at 0.1 C and nearly 90 mAh g^−1^ at 2 C. Importantly, when the current density was restored to 0.1 C, the capacity returned to its initial value of 280 mAh g^−1^, confirming the structural stability of the electrode during high-rate cycling.

Furthermore, Zhang and collaborators [64] reported a synthesis strategy that integrates sequential two-step acid treatment with high-temperature pyrolysis (Figure 5g). The optimized sample, A-2.25-6-T, exhibited a specific capacity of 342.4 mAh g^−1^ in Na-ion half-cells (Figure 5h) and preserved 89.7% of its capacity during long-term cycling at 0.3 A g^−1^. In complementary work, Ding and co-workers [65] explored bamboo-derived HC, leveraging the inherently high cellulose content of bamboo, which, upon carbonization, yields a dense closed-pore network advantageous for Na storage. Among the prepared materials, HCB-1400, obtained at 1400 °C, displayed superior electrochemical behavior, achieving 328.4 mAh g^−1^ at 30 mA g^−1^ with excellent stability in half-cell tests. These findings underscore the pivotal role of carbonization temperature in determining interlayer spacing, graphitization, and pore structure, all of which govern Na-ion storage properties. Importantly, full-cell assemblies employing HCB-1400 and Na_3_V_2_(PO_4_)_3_ retained 93% of their initial capacity after 200 cycles.

In summary, the findings affirm that two-step high-temperature pyrolysis constitutes an effective strategy for overcoming the performance limitations associated with HC synthesized via the conventional one-step method. The initial low-temperature carbonization of biomass precursors promotes the formation of a stable C matrix, which is subsequently optimized through a high-temperature treatment. This sequential process results in significant improvements in the material’s electrochemical properties.

#### 3.1.3. Summary of High-Temperature Pyrolysis

High-temperature pyrolysis is a commonly applied method for thermally decomposing biomass precursors in an O_2_-free atmosphere. The process is typically divided into single-step and two-step routes. In the single-step route, precursors undergo direct carbonization at high temperatures, which quickly produces HC but often yields materials with low porosity and modest electrochemical properties. By contrast, the two-step approach introduces an initial low-temperature carbonization prior to high-temperature treatment. Although this sequence requires more processing time, it allows fine-tuning of carbonization conditions, thereby optimizing interlayer spacing, lattice ordering, and pore development to improve energy storage capabilities. Nyquist plots from electrochemical impedance spectroscopy (EIS) are frequently analyzed using Randles-type equivalent circuit models, where the semicircle diameter reflects the charge-transfer resistance (R_ct_). For optimized BHC anodes, R_ct_ values typically fall within 50–200 Ω, indicating efficient Na^+^ transport and good interfacial kinetics. Moreover, long-term cycling tests in recent studies consistently demonstrate over 80% capacity retention after 500–2000 cycles, underscoring the structural stability and durability of BHC electrodes.

Overall, the two-step high-temperature pyrolysis strategy effectively addresses the structural and electrochemical drawbacks of the conventional one-step route. This improvement arises from the formation of a robust C framework during the pre-carbonization stage, which is subsequently refined under high-temperature treatment to enhance energy storage capabilities.

### 3.2. Hydrothermal Treatment

Hydrothermal pretreatment is a thermochemical process wherein materials are subjected to moderate temperatures and saturated pressure within an aqueous environment [66]. This technique is instrumental in modifying and optimizing the structural characteristics of HC materials and has become an integral component of thermochemical processing protocols [67].

**Figure 6:**
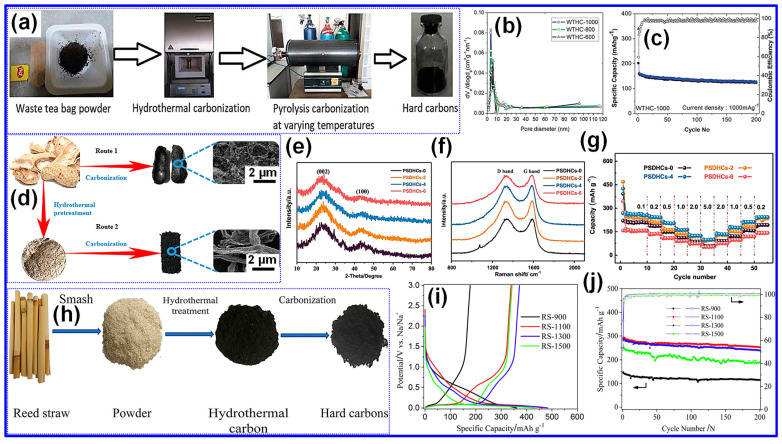
(**a**) Synthetic pathway for producing HC from spent tea bag powder. (**b**) Pore size analysis of WTHC-600, WTHC-800, and WTHC-1000. (**c**) Galvanostatic cycling profile of WTHC-1000 tested at 1000 mA g^−1^ from 0.01 to 2.0 V vs. Na/Na^+^. Adapted from [68]. Copyright 2019, Taylor & Francis. (**d**) Diagrammatic representation of the fabrication process for PSDHCs-x. (**e**) XRD, (**f**) Raman spectra, and (**g**) Rate behavior from 0.1 to 5.0 C of PSDHCs-x electrodes. Adapted from [69]. Copyright 2019, Elsevier B.V. (**h**) Visual overview of the preparation strategy for RS-x samples. (**i**) Initial GCD profiles, and (**j**) cycling stability of the RS-x electrodes. Adapted from [70]. Copyright 2018, Elsevier B.V.

Arie and colleagues [68] reported the synthesis of sheet-like HC through a combined route of hydrothermal treatment, physical activation, and subsequent high-temperature pyrolysis (Figure 6a). Using waste tea bag powder as the C precursor, the work underscored the promise of biomass waste as a renewable feedstock for SIB anodes. The hydrothermal step was particularly important, as it removed impurities such as lignin and hemicellulose, thereby generating a stable C framework for later activation. The obtained HC materials, designated WTHC-600, WTHC-800, and WTHC-1000, displayed a well-developed pore system (Figure 6b). Among them, WTHC-1000 showed the best electrochemical properties, maintaining a capacity of 127 mAh g^−1^ after 200 cycles in Na-ion half-cells (Figure 6c).

Meanwhile, Ren and colleagues [69] produced HC using peanut shells as the precursor through a combined hydrothermal and high-temperature pyrolysis approach (Figure 6d). The resulting products, labeled PSDHCs-x, where x denotes the hydrothermal treatment duration, showed distinct structural variations dependent on pretreatment time. As revealed by XRD patterns (Figure 6e), the d_002_ spacing expanded with longer hydrothermal treatment, promoting more efficient Na-ion intercalation. Raman spectra (Figure 6f) further demonstrated a reduced I*_D_*/I*_G_* ratio, indicative of an increase in defect sites that could enhance Na-ion adsorption. The optimized sample exhibited superior electrochemical behavior, with excellent cycling stability and rate performance (Figure 6g).

Shi’s team [70] prepared HC materials through hydrothermal treatment at 200 °C for 24 h followed by high-temperature pyrolysis (Figure 6h). Among the samples, RS-1300 achieved the best performance, delivering a reversible capacity of 372 mAh g^−1^ (Figure 6i) along with excellent cycling behavior (Figure 6j). In related work, Zhao and colleagues [71] utilized dried mango powder as the C source and introduced H_2_SO_4_ during the hydrothermal stage. The acidic medium promoted precursor depolymerization into monosaccharides, thereby enhancing carbonization efficiency. In addition, the self-assembly of monosaccharides into spherical particles with reduced surface tension contributed to the formation of a more uniform C microstructure.

Overall, hydrothermal treatment serves as an effective pretreatment route that promotes impurity removal, pore formation, and functional group introduction under mild conditions. This strategy enhances structural stability and facilitates Na^+^ transport, leading to improved cycling behavior. However, compared to direct pyrolysis, hydrothermal methods generally yield lower specific capacities unless coupled with subsequent activation or doping. The main lesson is that hydrothermal pretreatment is best employed as a complementary step rather than a stand-alone strategy, particularly for tailoring biomass precursors with complex compositions.

### 3.3. Activation Pretreatment

In HC synthesis, activation is generally achieved through either physical or chemical methods [72]. Physical activation consists of carbonizing the precursor under an inert atmosphere to remove non-C components, followed by treatment with oxidizing gases such as O_2_ or CO_2_ to induce porosity. In contrast, chemical activation involves impregnating the precursor with activating agents (e.g., KOH, H_3_PO_4_) before subjecting it to high-temperature carbonization. This approach offers several advantages, including lower activation temperatures, enhanced C yields, reduced processing times, and the formation of a highly porous structure. Nonetheless, it introduces challenges, such as the corrosiveness of the chemicals used and the requirement for extensive post-treatment to remove residual reagents.

Kumaresan’s team [73] prepared HC from the male inflorescence of *Borassus flabellifer* using a thermal activation route conducted under a mixed N_2_/CO_2_ atmosphere. When tested as an SIB anode, the material delivered a specific capacity of 358 mAh g^−1^. This enhanced behavior was attributed to the presence of fractured edge sites and a uniformly distributed nanoporous framework. The findings underscore the potential of this low-cost biomass source for producing defect-engineered HCs suitable for high-performance energy storage applications.

Prosini and coworkers [74] carried out a comparative investigation of HC materials activated with different alkali hydroxides, namely NaOH, KOH, RbOH, and CsOH. Electrochemical characterization showed clear variations in capacity among the prepared anodes, which were linked to both the type of wood precursor and the activating hydroxide used. Among the samples, the KOH-activated HC exhibited the highest capacity and superior rate capability. These findings were rationalized through a dual-action mechanism in which the alkali cations facilitated both the development of porosity and the expansion of interlayer spacing within the C matrix, with cation size playing a pivotal role in modulating activation efficiency.

**Figure 7:**
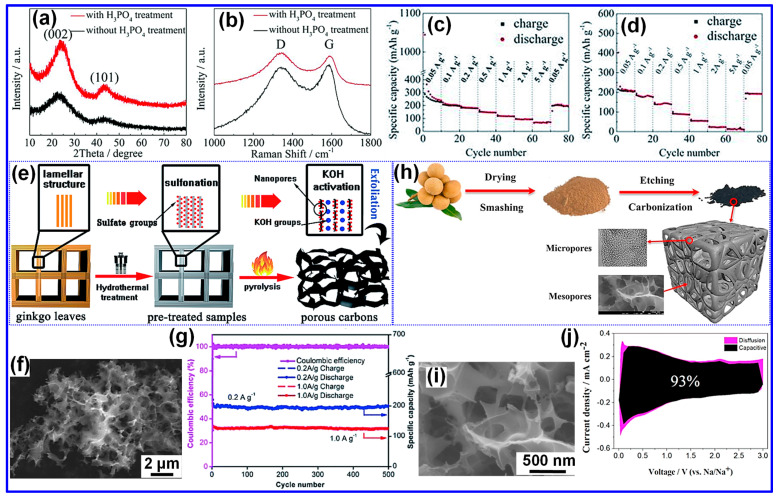
(**a**) XRD, and (**b**) Raman spectra of C materials obtained from pomelo peels, illustrating the impact of H_3_PO_4_ treatment. Rate capability of activated porous C: (**c**) with H_3_PO_4_, and (**d**) without H_3_PO_4_ treatment. Adapted from [75]. Copyright 2014, Royal Society of Chemistry. (**e**) Fabrication schematic of activated C produced from ginkgo leaf biomass. (**f**) SEM micrograph of ACGL. (**g**) Cycling stability of ACGL at 0.2 and 1 A g^−1^. Adapted from [79]. Copyright 2017, Royal Society of Chemistry. (**h**) Fabrication schematic of porous C produced from longan shells. (**i**) TEM micrograph, and (**j**) Capacitive charge storage contributions at a sweep rate of 1 mV s^−1^ of LPC-800. Adapted from [80]. Copyright 2018, Elsevier B.V.

Huang and colleagues [75] synthesized porous HC by treating pomelo peel with H_3_PO_4_, followed by a drying step. The resultant H_3_PO_4_-activated C exhibited a 3D, interconnected porous architecture. As illustrated in Figure 7a,b, XRD and Raman spectroscopy of both untreated and treated samples revealed broad diffraction features, with the activated material displaying an elevated *I_D_/I_G_* ratio. This increase signifies a higher degree of structural disorder, attributable to partial disruption of graphitic domains by the H_3_PO_4_ treatment. Electrochemical characterization (Figure 7c,d) demonstrated strong rate capability, with the material maintaining a reversible capacity of 71 mAh g^−1^ even at a current density of 5 A g^−1^.

Wu’s group [76] proposed a refined approach for preparing high-performance HC anodes for batteries, using bamboo powder as the C source. The method applied a two-step carbonization sequence combined with H_2_SO_4_ treatment at various stages: before carbonization (RHC), after pre-carbonization (PHC), and after final carbonization (SHC). An untreated control (UHC) was also prepared. Acid pretreatment promoted the breakdown of hemicellulose and the removal of residual impurities, producing C with short-range curved layers, open porosity, and oxygenated functional groups. These features enabled multiple Na storage mechanisms, including interlayer intercalation, pore filling, and surface adsorption, leading to excellent electrochemical behavior. Specifically, the RHC sample achieved a reversible capacity of 320.96 mAh g^−1^, an ICE of 91.45%, and a rate capability of 108.8 mAh g^−1^ at 8 A g^−1^, along with outstanding cycling stability.

Additionally, Wang and colleagues [77] investigated waste rosewood as a biomass precursor for fabricating HC anodes in SIBs. Their approach combined chemical pretreatment with NaClO_2_ and NaOH, used to remove lignin and hemicellulose, with subsequent pyrolysis at 1100 °C. The obtained HC displayed numerous closed pores with thin walls. Among the samples, ChT-1100 delivered the best electrochemical behavior, achieving a reversible capacity of 326 mAh g^−1^ at 20 mA g^−1^ and sustaining 230 mAh g^−1^ even under 5000 mA g^−1^. These enhancements were attributed to the structural refinement induced by pretreatment, which increased the number of accessible pore sites and facilitated efficient Na^+^ diffusion, thereby improving rate performance and cycling durability.

Xu and collaborators [78] employed natural lotus peduncle powder (LPP) as a C precursor and applied a microwave-assisted activation with H_2_SO_4_. The optimized HC demonstrated remarkable electrochemical behavior, achieving a reversible capacity of 213.3 mAh g^−1^ at 5 A g^−1^. In addition, the material showed excellent cycling durability, preserving 90.2% of its initial capacity after 2000 cycles at 1 A g^−1^.

In addition, Tian and coworkers [81] applied KOH activation to peanut shells, yielding porous HC materials that retained a specific capacity of 193 mAh g^−1^ after 400 cycles at a current density of 0.25 A g^−1^ in Na-ion half-cells. In a related effort, Hao’s team [79] employed ginkgo leaves as a biomass precursor (Figure 7e). The leaves were pretreated with H_2_SO_4_, blended with KOH in a 1:1 mass ratio, and carbonized to produce AC from ginkgo leaves (ACGLs). The resulting material exhibited a distinctive 3D porous structure with interconnected channels (Figure 7f). Electrochemical evaluation revealed that ACGL retained 200 mAh g^−1^ after 500 cycles, underscoring its strong cycling stability in Na-ion half-cells (Figure 7g).

Luo and collaborators [80] utilized longan shells as a biomass precursor for synthesizing HC, as depicted in Figure 7h. The raw material was mixed with KOH in a 1:3 mass ratio and subjected to carbonization, producing a unique HC sample (LPC-800) characterized by a well-developed micro- and mesoporous framework (Figure 7i). This architecture resulted in an exceptionally high SSA, which translated into a notable reversible capacity of 350.6 mAh g^−1^ at 0.1 A g^−1^. As shown in Figure 7j, the capacitive contribution increased with scan rate, reaching 93% at 1 mV s^−1^. The pronounced capacitive behavior highlights the abundance of accessible active sites for Na^+^ adsorption, which significantly improves both capacity and rate capability.

Activation is highly effective for generating porous structures and large surface areas, thereby increasing Na^+^ storage sites and improving rate capability. Chemical activation in particular offers fine control over porosity but requires corrosive reagents and post-treatment to remove residues, while physical activation is less aggressive but provides limited pore development. The trade-off, therefore, involves balancing enhanced surface activity and conductivity with reduced ICE and potential processing challenges. The key takeaway is that activation should be strategically applied to maximize pore accessibility without excessively sacrificing ICE, making it a versatile but carefully tuned pretreatment method.

### 3.4. Heteroatom Doping

Heteroatom doping has emerged as an efficient pretreatment strategy for enhancing the electrochemical kinetics of HC anodes by facilitating both Na-ion diffusion and electron transport within the C matrix [82]. This modification not only raises the number of electroactive sites but also contributes to the development of a more porous C structure. Among various doping strategies, single-element doping and co-doping are the most prevalent. Nitrogen is frequently selected as the dopant because many biomass feedstocks naturally contain protein-derived N-species. As such, heteroatom doping offers a practical and scalable route for engineering atomically modified HC materials.

Senthil and coworkers [83] utilized seaweed as a renewable C source to obtain N-enriched, self-doped HC with a well-developed pore network. The optimized sample delivered a capacity of 303 mAh g^−1^ after 100 cycles at 100 mA g^−1^. Similarly, Yan’s team [84] produced N-doped C sheets (NDCSs) from okara, achieving a N content of 9.89%. TEM revealed loosely stacked graphitic layers that provided abundant Na-ion intercalation sites, while microcrystalline regions enhanced electron transfer during redox processes. In a related investigation, Zhu’s group [85] synthesized porous HC by treating leaf biomass with H_3_PO_4_ (3:1 ratio) and thermally processing the mixture at 500 °C for 1 h. The resulting material featured a highly porous architecture, improving electrolyte access and charge transport. P doping further expanded the interlayer spacing, enhancing Na^+^ adsorption and overall storage efficiency.

**Figure 8:**
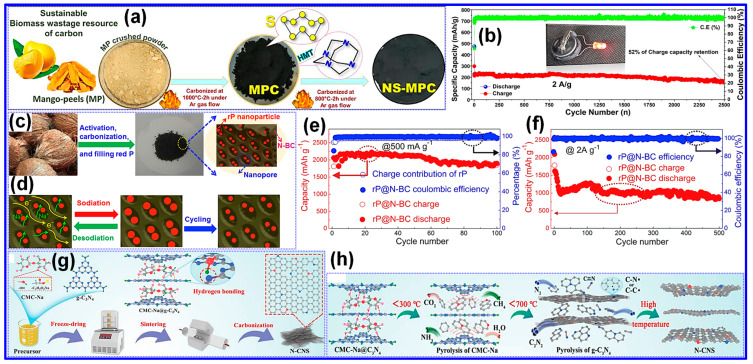
(**a**) Illustration of the synthetic route for mango peel-derived HC (MPC) and N/S co-doped HC (NS-MPC) via a straightforward carbonization. (**b**) Cycling stability of NS-MPC cell at a high current rate, with inset showing the LED device powered by the cell after 2500 cycles. Adapted from [86]. Copyright 2022, Elsevier B.V. (**c**,**d**) Pictorial demonstration of the rP@N-BC composite synthesis, along with optical images depicting the transformation from coconut shell biomass to rP@N-BC. Also shown is a schematic representation of the volume variation in rP particles during sodiation/desodiation within the composite matrix. Cycle stability and CE of rP@N-BC electrode at (**e**) 500 mA g^−1^, and (**f**) 2 A g^−1^. Adapted from [87]. Copyright 2019, Elsevier B.V. (**g**) Illustration detailing the synthesis pathway for N-CNS. (**h**) Evolution of structural features in the construction process of N-CNS. Adapted from [89]. Copyright 2023, Wiley VCH.

To evaluate whether co-doping with multiple heteroatoms offers synergistic benefits over single-element doping, several research efforts have been conducted. In one such study, Liu and coworkers [86] synthesized N,S co-doped C (NS-MPC) using mango peel as the C source, S powder as the S dopant, and hexamethylenetetramine as the N precursor. The mixture was preheated at 350 °C and subsequently carbonized at 800 °C for 2 h under Ar, producing the NS-MPC (Figure 8a). The material demonstrated remarkable durability, retaining a reversible capacity of 155 mAh g^−1^ after 2500 cycles at 2 A g^−1^ (Figure 8b).

He and colleagues [87] employed N-rich coconut shells as biomass precursors to prepare NPC (rP@N-BC) (Figure 8c,d). The optimized sample delivered an impressive capacity of 1857 mAh g^−1^ after 100 cycles with a minimal fade rate of 0.07% per cycle (Figure 8e). Even after 500 cycles, a high reversible capacity of 845 mAh g^−1^ was retained (Figure 8f), confirming its outstanding durability. In a parallel study, Yuan’s group [88] fabricated N,S co-doped HC (N,S-HC) via one-step pyrolysis of walnut shells. The natural S content in the walnut shells facilitated in situ S-doping, while co-doping with N and S effectively enlarged interlayer spacing and significantly enhanced Na storage capacity.

Elemental doping influences the performance of HC anodes in SIBs with varying effects. Moderate doping levels are generally beneficial, enhancing electrochemical properties, while excessive doping may be counterproductive. For instance, N doping improves conductivity and introduces active sites for Na^+^ storage. However, high N content can disrupt localized graphitic domains, impairing conductivity. Among N species, pyrrolic N is particularly advantageous as it enlarges interlayer spacing and lowers the Na^+^ insertion energy barrier due to its out-of-plane electron lone pairs. Both pyridinic and pyrrolic N contribute to increased active site density, thereby enhancing specific capacity.

Zhao and collaborators [89] developed N-doped HC (N-HC) from Na carboxymethyl cellulose (CMC-Na), employing g-C_3_N_4_ as a catalytic agent. The resulting material (Figure 8g,h) featured expanded interlayer spacing and abundant defects. During pyrolysis, nitrile intermediates were converted to C-N/C-C radicals, facilitating NSs-like architectures. This tailored architecture achieved excellent electrochemical behavior, including 192.8 mAh g^−1^ at 5 A g^−1^ and stable cycling with 233.3 mAh g^−1^ retained after 2000 cycles at 0.5 A g^−1^, offering a deeper mechanistic understanding of Na storage in C anodes.

Heteroatom doping is a powerful approach to modify the electronic structure of HC, expand interlayer spacing, and introduce abundant electroactive sites. N-doping, in particular, enhances conductivity and lowers the Na^+^ insertion barrier, while co-doping with elements such as S or P provides synergistic benefits. However, excessive doping can disrupt graphitic domains, lowering conductivity and structural stability. Thus, the trade-off lies in determining the optimal doping level and species for targeted improvements. The main lesson is that controlled, moderate heteroatom doping offers a highly effective route to boost both capacity and cycling stability of BHC anodes.

### 3.5. Template Pretreatment

Template pretreatment methods can be generally categorized into soft and hard templating strategies. Soft template strategy utilizes amphiphilic agents that self-assemble into micelles or reverse microemulsions, which are subsequently decomposed during pyrolysis to form well-organized porous C structures. Conversely, hard templating utilizes rigid, solid materials as scaffolds for monomer deposition and polymerization. After thermal treatment and template removal, commonly by chemical etching or calcination, a porous C matrix is obtained with precise structural control.

**Figure 9:**
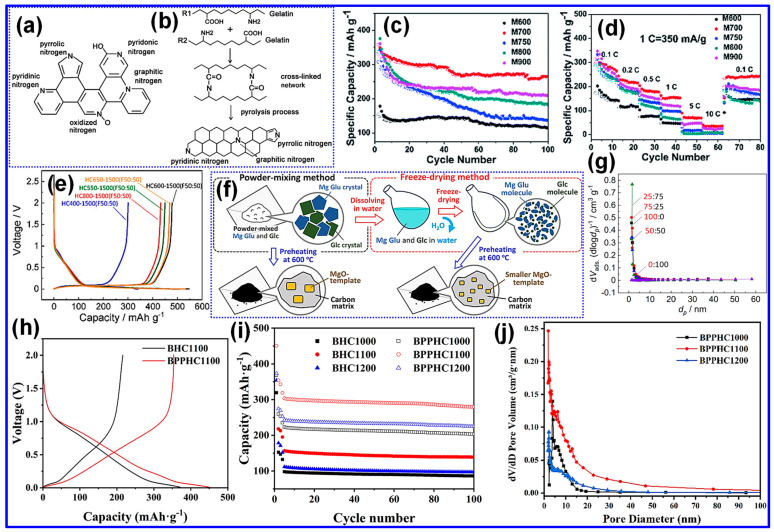
(**a**,**b**) Proposed N configurations in gelatin-derived C and the corresponding thermal cross-linking and pyrolysis pathways of gelatin molecules. (**c**,**d**) Cycle and rate capabilities of M600-M900. Adapted from [90]. Copyright 2015, Royal Society of Chemistry. (**e**) First GCD plots of HC400-1500 (F50:50), HC550-1500 (F50:50), HC600-1500 (F50:50), HC650-1500 (F50:50) and HC800-1500 (F50:50). (**f**) Pictorial representation of two distinct methods employed for mixing Mg Glu and Glc precursors. (**g**) BJH curves of HC600-1500(F100:0), HC600-1500(F75:25), HC600-1500(F50:50), HC600-1500(F25:75), and HC600-1500(F0:100). Adapted from [91]. Copyright 2021, Wiley VCH. (**h**) Initial GCD curves of BHC1100 and BPPHC1100. (**i**) Comparative cycling performance of BHC and BPPHC materials pyrolyzed at 1000, 1100, and 1200 °C under a current density of 1 C. (**j**) Pore size distribution profiles of BPPHC samples treated at 1000, 1100, and 1200 °C. Adapted from [92]. Copyright 2024, Wiley VCH.

Guan and coworkers [90] synthesized N-doped mesoporous HC by co-pyrolyzing gelatin with Mg citrate at 600–900 °C. Mesopores were successfully formed even at lower temperatures, producing a more uniform pore distribution than the original precursors. As illustrated in Figure 9a,b, the C matrix incorporated N in pyridinic, pyrrolic, and oxidized forms, all of which enhanced Na storage. Electrochemical evaluation (Figure 9c,d) demonstrated that the materials (M600–M900) delivered excellent rate capability and cycling durability in SIBs.

Kamiyama and colleagues [91] synthesized HC using a MgO-templated method. The optimized HC600-1500 (F50:50) sample delivered excellent electrochemical properties, achieving 478 mAh g^−1^ reversible capacity and 88% ICE (Figure 9e). As illustrated in Figure 9f, Mg gluconate (Mg Glu) and glucose (Glc) were uniformly dispersed through freeze-drying, after which preheating at 600 °C produced MgO nanocrystals that acted as templates. Final carbonization at 1500 °C generated nanoscale pores. The combined effect of abundant nanopores and expanded graphitic layers (Figure 9g) was responsible for the enhanced Na storage.

Likewise, Yin’s group [92] prepared HC from bamboo powder through chemical activation and dual-template pretreatment with Pluronic F127 and Mg(CH_3_COO)_2_·4H_2_O, followed by pyrolysis at different temperatures. The BPPHC1100 sample, obtained at 1100 °C, showed the best performance, delivering 354 mAh g^−1^ at 0.2 C and retaining 92% capacity after 100 cycles (Figure 9h,i). The improved performance was attributed to the abundant nanoporous architecture and expanded interlayer spacing, both induced by the combined effects of chemical activation and templating, as illustrated in Figure 9j. These synergistic modifications facilitated stable structural integrity and long-term cycling durability.

Template-assisted methods provide precise control over pore size distribution and architecture, enabling the design of highly ordered and uniform porous C. These materials often demonstrate superior rate capability and long-term cycling stability. However, the complexity, cost, and multistep nature of template removal limit scalability and practical deployment. The trade-off is between excellent structural tunability and the economic/environmental feasibility of large-scale production. The key lesson is that template pretreatment is most promising when applied to specialized high-performance applications, but further innovation is needed to simplify the process for industrial adoption.

## 4. Conclusions and Future Perspectives

### 4.1. Conclusions

The Na storage performance of HC anodes is closely governed by their structural characteristics, which are, in turn, largely dictated by the choice of biomass precursor. For instance, C materials that retain the native porosity of the source biomass tend to exhibit superior ion diffusion pathways and enhanced specific capacities [57]. However, the attainment of high-performance HC anodes necessitates meticulous optimization of processing conditions, particularly pretreatment strategies and pyrolysis temperatures. While elevated pyrolysis temperatures generally diminish surface defects, pore volume, and SSA, thereby enhancing ICE [62,63]. They simultaneously induce a contraction in interlayer spacing, thereby raising the energy barrier for Na intercalation [93]. Crucially, when the interlayer distance falls below 0.36 nm, Na ions experience considerable difficulty in intercalating, which adversely affects the plateau capacity [94].

Building upon the preceding discussion, this review systematically examines diverse synthesis strategies for BHC, each characterized by unique benefits and limitations. One-step high-temperature pyrolysis, while energy-intensive, offers operational simplicity and satisfactory electrochemical performance, making it a viable candidate for large-scale deployment. In contrast, the two-step pyrolysis method, despite its increased complexity, affords superior structural modulation. Specifically, the constrained atomic rearrangement during single-step treatment typically yields pseudo-graphitic C domains. Conversely, the sequential approach promotes the formation of quasi-equilibrium hexagonal C nanodomains embedded within an amorphous matrix [95], thereby contributing to enhanced ICE, improved reversible capacity, and superior rate capability.

Moreover, hydrothermal pretreatment not only demonstrates environmental compatibility but also enhances electrochemical capacity and stability. Compared to direct pyrolysis, it promotes the formation of larger nanopores, which facilitate improved Na ion transport and storage [96]. Additionally, activation pretreatment is employed to tailor the HC structure by introducing further porosity [97] and increasing the SSA while promoting a more ordered arrangement [98]. Furthermore, heteroatom doping serves as an effective means to expand interlayer spacing and fine-tune material properties, thereby significantly enhancing capacity and cycling stability [99]. Although template pretreatment is the most intricate among these approaches, it offers precise structural control over the resulting HC [91].

**Figure 10:**
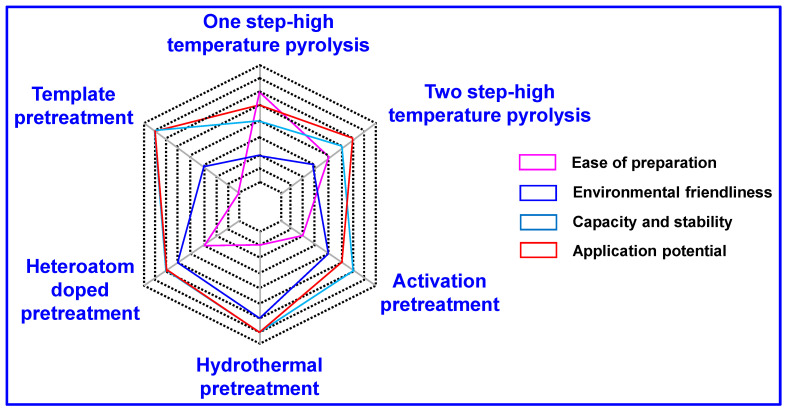
A critical evaluation of the advantages and limitations associated with the six principal synthesis strategies.

In conclusion, each of the aforementioned preparation strategies offers distinct advantages and limitations concerning synthesis complexity, environmental sustainability, electrochemical capacity, structural stability, and scalability. The optimal selection depends on the intended application and specific material requirements. A comparative overview of the advantages and disadvantages of the six primary methods is presented in Figure 10, while Table 1 compiles the electrochemical performance metrics of HC derived from these strategies for use in SIBs. These insights lay the groundwork for future advancements in anode material development and practical implementation in SIBs.

### 4.2. Future Perspectives

**Figure 11:**
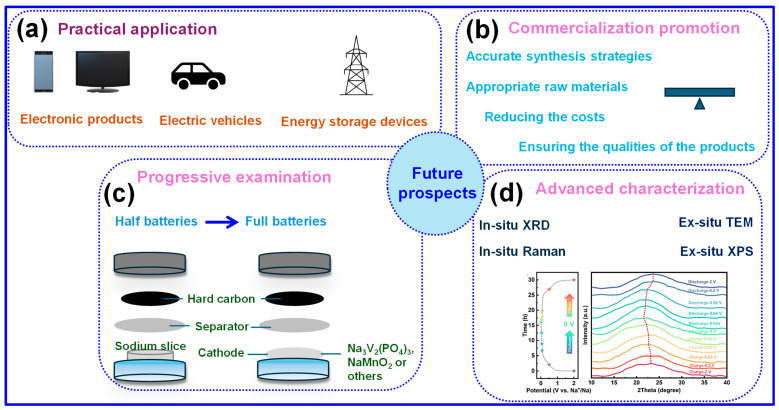
Future perspectives for the application of BHC materials in SIBs include: (**a**) Real-world deployment. (**b**) Acceleration of commercialization efforts. (**c**) Systematic performance evaluation. (**d**) In-depth structural and electrochemical characterization. Adapted from [104]. Copyright 2024, Elsevier B.V.

In recent years, significant progress in electrode material design has markedly advanced the development of SIBs, as illustrated in Figure 11a. These scientific and technological strides are progressively transitioning into commercial implementation, thereby catalyzing large-scale SIB manufacturing and offering a strategic advantage across the battery supply chain. Among the various anodes under investigation, HC has emerged as a leading candidate due to its low cost and high theoretical capacity, playing an instrumental role in the industrialization of SIBs [100,101]. In particular, BHC is garnering increasing attention for its environmental sustainability, economic feasibility, and resource abundance. Notably, China possesses vast reserves of agricultural and forestry biomass, estimated at nearly one billion tons annually, yet over 80% of this potential remains underutilized or wasted through burning. Thus, the effective valorization of biomass resources represents a critical direction for both sustainable development and energy technology innovation.

In efforts to commercialize BHC for energy storage applications, Kuraray, a Japanese corporation, employed coconut shells from Southeast Asia as the primary precursor. The resulting HC exhibited commendable electrochemical characteristics, including a capacity of approximately 300 mAh g^−1^ and an ICE exceeding 80%. Nevertheless, the complexity and cost of the production process rendered it unsuitable for large-scale manufacturing. Parallel initiatives by various Chinese companies, which utilized alternative biomass feedstocks, have also faced considerable challenges. These include technical bottlenecks and inadequate performance, particularly with regard to achieving the long-term cycling stability essential for practical SIB deployment.

To enable the successful commercialization of HC anodes for SIBs, the implementation of advanced material engineering strategies is essential to enhance both specific capacity and cycling durability. Unlike LIBs, SIBs face rate performance constraints largely due to the comparatively larger ionic radius of Na^+^, which impedes intercalation kinetics. Moreover, the stability of long-term cycling is closely tied to the SEI. The increased reactivity between Na^+^ ions and the electrolyte can lead to excessive SEI growth or mechanical degradation (e.g., cracking), thereby compromising electrochemical performance and lifespan. Compounding this issue is the inherently poor electrical conductivity of unmodified HC, which contributes to elevated internal resistance during operation. A promising solution lies in the development of composite electrodes that exhibit improved conductivity and structural robustness.

Another promising strategy for enhancing the microstructure of HC at the nanoscale involves the incorporation of metallic ash elements [102], which can significantly contribute to the development of high-performance SIBs. For instance, potassium (K) release during pyrolysis can generate pore channels that increase Na ion storage capacity. Additionally, thermal competition between K atoms and C layers slows the rearrangement of C atoms, thereby reducing graphitization, preserving wider interlayer spacing, and promoting Na ion diffusion [103]. Furthermore, optimizing raw material selection and manufacturing routes is crucial for reducing production costs without compromising product quality, as illustrated in Figure 11b.

While substantial progress has been made in the development of Na-ion half-cells, future investigations should shift towards Na-ion full cells using HC anodes (Figure 11c), as they provide a more comprehensive evaluation of battery performance by accounting for cathode-anode interactions. For instance, Zhang and coworkers [105] highlighted the economic and practical advantages of cathode material recovery in full-cell systems. Moreover, advanced characterization techniques (Figure 11d) should be prioritized to deepen understanding of interfacial and structural phenomena. As the SIB market expands, continued research across anode materials, electrolytes, and cathodes will be critical for enabling scalable, low-cost, and environmentally sustainable SIB technologies. Given China’s increasingly stringent environmental regulations and emphasis on energy conservation, BHC is poised for broader industrial adoption. Realizing this potential will require coordinated efforts across multiple sectors to foster an integrated industrial ecosystem aligned with the “Made in China 2025” strategic initiative.

Looking forward, the scalability and industrial implementation of BHC remain crucial for the widespread adoption of SIBs. The use of abundant agricultural and forestry residues offers a sustainable route for large-scale production, provided that synthesis processes can be optimized for cost-efficiency and reproducibility. Future work should focus on improving electrode packing density, interfacial stability, and compatibility with high-voltage electrolytes to enable integration into commercial full-cell configurations. Collaborative efforts between academia and industry will be essential to transition these materials from laboratory demonstrations to reliable, market-ready energy storage technologies.

## Figures and Tables

**Table 1:** Comparative summary of the electrochemical properties of HC anodes produced through different synthesis strategies for SIBs.

Preparation Approach	Anode Material	Specific Capacity (mAh g^−1^) [Current Density (A g^−1^/C-Rate)]	Cycle Stability (%) [CURRENT Density (A g^−1^/C-Rate)]	Number of Cycles	Ref.
High-temperature pyrolysis	HCC1300	298 [0.03 A g^−1^]	97 [0.2 C]	100	[58]
	CC-1600	358 [0.03 A g^−1^]	71 [2 C]	2000	[62]
	SP-1200	430 [0.03 A g^−1^]	97.5 [0.05 A g^−1^]	200	[35]
	A-2.25-6-T	342 [0.03 A g^−1^]	88.5 [0.3 A g^−1^]	400	[64]
	LS1200	329 [0.05 A g^−1^]	89.7 [0.05 A g^−1^]	200	[59]
	CDHC-1300	311 [0.03 A g^−1^]	83.5 [0.1 C]	1000	[63]
Hydrothermal pretreatment	PSDHCs-4	256 [0.1 C]	97 [0.1 C]	100	[69]
	WTHC-1000	375 [0.01 A g^−1^]	81 [0.1 A g^−1^]	100	[68]
	RS-1300	372 [0.1 C]	84 [0.4 C]	200	[70]
Activation pretreatment	LPC-800	351 [0.1 A g^−1^]	85.4 [0.2 A g^−1^]	50	[80]
	H_3_PO_4_-AC	288 [0.05 A g^−1^]	84.6 [0.2 A g^−1^]	220	[75]
	ACGL	320 [5 A g^−1^]	99 [0.2 A g^−1^]	500	[79]
Heteroatom-doped pretreatment	rP@N-BC	248 [0.05 A g^−1^]	74.8 [0.5 A g^−1^]	100	[87]
	NS-MPC	400 [0.1 A g^−1^]	52 [2 A g^−1^]	2500	[86]
	N-CNS-1050	305 [0.05 A g^−1^]	76.5 [0.5 A g^−1^]	2000	[89]
Template pretreatment	BPPHC1100	354 [0.05 A g^−1^]	92 [1 C]	100	[92]
	M750	360 [0.1 A g^−1^]	51 [0.1 A g^−1^]	100	[90]
	HC600-1500(F50:50)	478 [0.025 A g^−1^]	96 [0.25 A g^−1^]	25	[91]

## Data Availability

Not applicable.
